# DeepGenePrior: A deep learning model for prioritizing genes affected by copy number variants

**DOI:** 10.1371/journal.pcbi.1011249

**Published:** 2023-07-24

**Authors:** Zahra Rahaie, Hamid R. Rabiee, Hamid Alinejad-Rokny

**Affiliations:** 1 BCB Group, DML, Department of Computer Engineering, Sharif University of Technology, Tehran, Iran; 2 UNSW Biomedical Machine Learning Lab (BML), the Graduate School of Biomedical Engineering, UNSW Sydney, Sydney, Australia; University of Washington, UNITED STATES

## Abstract

The genetic etiology of brain disorders is highly heterogeneous, characterized by abnormalities in the development of the central nervous system that lead to diminished physical or intellectual capabilities. The process of determining which gene drives disease, known as “gene prioritization,” is not entirely understood. Genome-wide searches for gene-disease associations are still underdeveloped due to reliance on previous discoveries and evidence sources with false positive or negative relations. This paper introduces DeepGenePrior, a model based on deep neural networks that prioritizes candidate genes in genetic diseases. Using the well-studied Variational AutoEncoder (VAE), we developed a score to measure the impact of genes on target diseases. Unlike other methods that use prior data to select candidate genes, based on the "guilt by association" principle and auxiliary data sources like protein networks, our study exclusively employs copy number variants (CNVs) for gene prioritization. By analyzing CNVs from 74,811 individuals with autism, schizophrenia, and developmental delay, we identified genes that best distinguish cases from controls. Our findings indicate a 12% increase in fold enrichment in brain-expressed genes compared to previous studies and a 15% increase in genes associated with mouse nervous system phenotypes. Furthermore, we identified common deletions in *ZDHHC8*, *DGCR5*, and *CATG00000022283* among the top genes related to all three disorders, suggesting a common etiology among these clinically distinct conditions. DeepGenePrior is publicly available online at http://git.dml.ir/z_rahaie/DGP to address obstacles in existing gene prioritization studies identifying candidate genes.

## Author summary

DeepGenePrior is a deep learning-based method for prioritizing genes in genetic diseases. Conventional tools utilize the guilt by association principle, which relies on prior knowledge to identify novel genes. In contrast, our method does not use any prior information. Furthermore, other tools rely on auxiliary data, including false positive or negative relations, which may lead to erroneous associations. Another group of methods relies on hypothesis testing, and fundamental issues regarding this group have been widely discussed in different papers.

We compared the results of DeepGenePrior with both statistical and machine learning studies against biological and classification benchmarks. Our method’s results outperformed current works in three brain disorders: autism, schizophrenia, and developmental delay.

## Introduction

Brain Disorders (BD) [1] are a group of disorders that affect the development of the nervous system, leading to dysfunctional brain functions that can influence memory, emotion, and learning ability. Well-studied loci associated with autism (a type of BD) include deletions in 16p11.2 [2–4] and duplications in 15q3 [5,6]. Genetic factors related to autism include TBX1 (involved in the regulation of development and associated with the 22q11.2 deletion syndrome), SHANK3 (a synaptic scaffolding gene), NLGN4 (a neuroligin gene), PCDH10 (a protocadherin gene), and NHE9 [7,8]. Other genes such as NRXN1, SHANK2, CNTN4, CNTNAP2, DPYD, DPP6, RFWD2, NLGN1, ASTN2, SYNGAP1, and DLGAP2, as well as DDX53-PTCHD1, are candidate genes for autism.

Schizophrenia (SCZ) is another disorder under the umbrella of brain disorders. CNVs disrupt several genes associated with SCZ, including TBX1 (also associated with autism), ERBB4 (encodes a receptor for NDF/heregulin), SLC1A3 (a glutamate transporter), RAPGEF4 (a nucleotide exchange factor), and CIT (a neuronal Rho-target gene) [7,8]. 7q11.2 and 15q13.3 have been reported as associated with SCZ [9]. In SCZ, a large (3 Mb) deletion on chromosome 22q11.21 is a significant risk factor [10], and other loci, including deletions at 1q21.1, deletions at 3q29, duplications of 16p11.2, deletions at 15q13.3, exonic deletions at 2p16.3, and duplications at 7q36.3, have also been reported [10]. Deletions in 1q24 (including the FMO group of genes and DNM3), 2q33.1 (SATB2), and 2p16.1 (NRXN1) are well-known variations associated with developmental delay (DD) [11].

Research on the genetics of diseases has implications for diagnosing, treating, and developing drugs for these disorders. Understanding the genetic etiology of brain disorders can provide valuable insights into effective prevention and treatment methods. Gene prioritization, the process of identifying genes that most likely contribute to a disease or phenotype, can be used in BDs. This work uses case and control copy number variants as input to prioritize causal genes associated with BDs.

The prioritization of genes relies on various types of evidence. According to [12], gene-disease associations are grouped into five categories, namely functional, cross-species, same-compartment, mutation, and textual. The first category examines molecule interactions [13], while the second category discusses homolog genes that cause similar phenotypes in other organisms [14]. Same-compartment evidence is based on the fact that the gene is involved in known disease-associated pathways or compartments, such as the cell membrane or nucleus [15]. Mutation evidence is based on Single Nucleotide Polymorphism (SNP) and structural variants, which is also the focus of this study [16]. Text evidence can be obtained from online collections like PubMed [17].

Several gene prioritization methods have been reviewed in [18–21], and from a methodological point of view, they can be classified into statistical and machine learning methods. The first group primarily employs hypothesis testing, such as exact tests like Fisher’s or permutation tests, to determine whether a gene is associated or not. However, several studies have reported p-value fallacies, such as distributional assumptions, limitations in data collection, and misleading results [22]. In addition, power loss and dependent values are discussed in detail as other criticisms of marginal p-values in [3]. Other issues can arise with these types of analyses, such as not considering all the heterogeneous features of genes.

Machine learning (ML) methods often rely on the ’guilt by association’ (GBA) principle [23–25]. This principle suggests that the new genes associated with a disease interact with the most recently discovered genes in a network that encodes similarities between genes. Inference of different types of networks can then lead to the discovery of new genes. In other words, ML methods require seed data (in this case, genes that implicitly characterize the disorder) [18] and a similarity metric to determine which candidate genes are similar or associated with the seed(s). However, issues arise with this approach, as discussed in [23,24]. For instance, it is impossible to discover a novel gene association that does not relate to the previous ones. Additionally, genes of a novel genetic disease with unknown roots cannot be found [26,27] due to the dependency of these methods on prior information.

**Fig 1:**
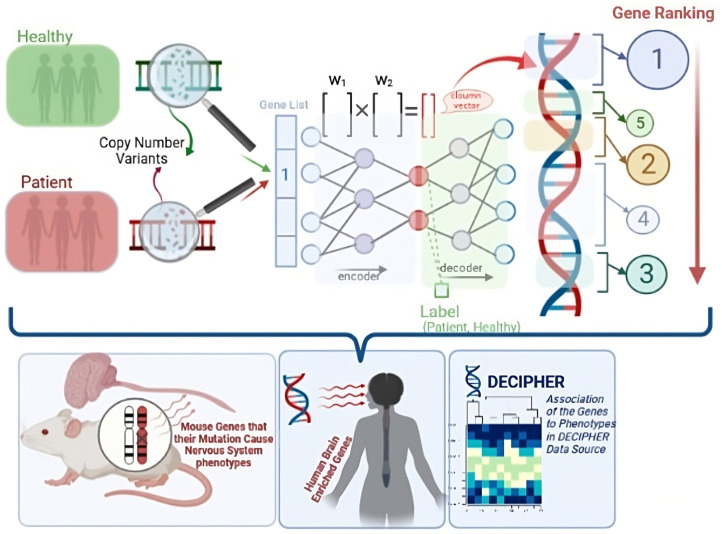
Summary of the Method and Analyses of the Results (Created with BioRender.com). A deep learning model learns the distinctions between cases and controls; then, the learned weights are used to prioritize the genes. After training, the results are evaluated using mutant mouse genes, human brain-enriched genes, DECIPHER data, and gene ontology analyses.

The issues discussed above hinder an ideal gene prioritization solution. To overcome these issues, we propose the DeepGenePrior method, which falls in the fourth category suggested in [12], as a deep learning architecture for gene prioritization. DeepGenePrior uses the well-studied autoencoder architecture with a variational learning framework. The Variational AutoEncoder [28,29] (VAE) is the stochastic variant of the autoencoder. Our method uses Copy Number Variants (CNV) data for gene prioritization. We train the network of neurons with all CNVs of cases and controls for all three diseases, followed by fine-tuning with the CNVs of the target disease. Controls and cases have zero and one CNV labels for the supervised learning phase. Finally, we build a score for every gene using the network weights and prioritize them. Fig 1 summarizes the method.

Our proposed method addresses gaps in previous studies and offers several advantages. First, it does not rely on theoretical assumptions like those in the hypothesis testing. Second, it does not require seed data, which is needed for methods based on guilt by association. Third, it does not rely on networks with false relations, like protein-protein networks.

We used CNVs from brain disorders to evaluate our method and compared them against major tools. We identified significantly mutated genes and found that our method detects genes that are 12% more enriched in brain expression than other tools. Furthermore, we compared the detected genes to those that cause nervous system phenotypes in mice and found our results to be 15% more enriched than other methods.

In addition, we examined genes that were exclusively overrepresented in one gender and analyzed the relationships between the detected genes and various phenotypes in the DECIPHER data source and the gene ontology of the putative genes. We found three genes common among the top genes associated with all three diseases: *ZDHHC8*, *DGCR5*, and *CATG00000022283*. According to the literature [30], defects found in *ZDHHC8* can be linked to susceptibility to schizophrenia. Also, we found that deletions in *CYFIP1*, *PRODH*, *XXBAC*, *B444P24*, *LINC00896*, *ZDHHC8*, *AC006547*, *NIPA2*, *RTN4R*, *NIPA1*, and *TUBGCP5* are associated with schizophrenia and developmental delay.

The following section describes our algorithm, the data we used, and the experiments we conducted. We then discuss our results before presenting our conclusions and future work in the final section.

## Results

### Prioritization of the genes in BDs

A deep learning model was utilized to identify the genes associated with BDs. The model was trained using copy number variants (CNVs) from all cases and controls, and the resulting model weights were employed to determine scores. The UCSC Lift Genome Annotations [31] tool was employed to convert all CNVs to the hg19 genome, and the locations of all CNVs were confirmed using NCBI remap tools [32]. CNVs smaller than one kilobase pair were excluded from the analysis.

The study showcases Tables 1, 2, and 3, displaying the top 40 genes for each disorder, accompanied by their respective p-values. Table 4 illustrates the methodology employed for Fisher’s exact test. Specifically, CaseOV represents the overlaps between cases and the genes, while ControlOV represents the overlaps between controls and the genes.

**Table 1:** Top 40 genes associated with developmental delay are presented. The model’s top findings on the developmental delay (DD) data source are reported herein. Each row provides information on gene names, overlapping cases and controls, P-value, and the type of genetic variation.

Gene name	Status	P-Value	CaseOV	ControlOV	Gene name	Status	P-Value	CaseOV	ControlOV
*TDRP*	dup	5.19E-159	293	18	*NANOG*	del	6.13E-122	221	12
*DGCR5*	del	2.17E-159	288	15	*SLC2A14*	del	6.13E-122	221	12
*PRODH*	del	1.46E-154	282	16	*CYFIP1*	del	5.85E-118	230	21
*LCE3E*	del	1.51E-153	257	5	*NIPA2*	del	5.85E-118	230	21
*ERICH1*	dup	5.32E-140	275	26	*TUBGCP5*	del	1.31E-117	231	22
*CATG0101427*	dup	1.97E-143	260	14	*NIPA1*	del	5.85E-118	230	21
*ERICH1-AS1*	dup	2.72E-147	254	8	*CATG0022283*	del	5.85E-118	230	21
*RP11-462G22*	dup	3.88E-150	249	4	*CATG0022286*	del	5.85E-118	230	21
*CATG0074892*	dup	1.64E-149	248	4	*CATG0024378*	del	5.85E-118	230	21
*CATG0074890*	dup	6.38E-148	248	5	*SLC2A3*	del	8.47E-121	221	13
*CATG0074891*	dup	6.38E-148	248	5	*CATG0007863*	del	1.02E-120	219	12
*RP11-462G22*	dup	6.38E-148	248	5	*CATG0011162*	del	2.79E-118	215	12
*CATG0074887*	dup	2.69E-147	247	5	*CATG0024374*	del	2.85E-112	222	22
*CATG0101432*	dup	6.15E-138	251	14	*LCE3D*	del	1.40E-105	256	57
*NPHP1*	dup	5.77E-133	260	24	*RTN4R*	del	3.99E-116	183	0
*MALL*	dup	1.29E-132	261	25	*ZDHHC8*	del	3.99E-116	183	0
*RP11-378A12*	dup	6.89E-135	246	14	*AC006547*	del	3.99E-116	183	0
*RP11-134O21*	dup	9.55E-125	243	22	*LINC00896*	del	3.99E-116	183	0
*CATG0117958*	dup	2.42E-124	234	17	*XXBAC-B444P24*	del	3.99E-116	183	0
*TMEM72-AS1*	dup	1.68E-123	236	19	*OTUD7A*	dup	3.29E-97	228	46

**Table 2:** Top 40 genes associated with schizophrenia are identified and presented. The model was trained using the schizophrenia data source, and the top results are reported herein. Each gene entry includes information on case and control overlaps, type of genetic variation, and corresponding P-value.

Gene Name	Status	P-value	CaseOV	ControlOV	Gene Name	Status	P-value	CaseOV	ControlOV
*DGCR6*	del	1.79E-06	129	60	*MED15*	del	1.79E-16	59	1
*PRODH*	del	7.19E-06	130	64	*DGCR8*	del	1.17E-17	58	0
*DGCR5*	del	7.19E-06	130	64	*CATG0058213*	del	1.17E-17	58	0
*AC009133*	dup	2.11E-14	66	5	*TMEM219*	dup	6.54E-14	61	4
*MVP*	dup	1.03E-14	64	4	*PTPRT*	del	2.77E-05	110	53
*CDIPT*	dup	1.03E-14	64	4	*CATG0058203*	del	2.31E-17	57	0
*SEZ6L2*	dup	1.03E-14	64	4	*CATG0058206*	del	2.31E-17	57	0
*CATG0027072*	dup	1.03E-14	64	4	*CATG0058209*	del	2.31E-17	57	0
*CDIPT-AS1*	dup	1.03E-14	64	4	*CLTCL1*	del	2.31E-17	57	0
*ASPHD1*	dup	1.90E-14	63	4	*COMT*	del	3.47E-16	58	1
*TRMT2A*	del	5.97E-18	59	0	*NIPA2*	del	0.00012	97	48
*RANBP1*	del	5.97E-18	59	0	*NIPA1*	del	0.00012	97	48
*ZDHHC8*	del	5.97E-18	59	0	*CATG0022283*	del	0.00012	97	48
*AC006547*	del	5.97E-18	59	0	*CATG0022286*	del	0.00012	97	48
*LINC00896*	del	5.97E-18	59	0	*CYFIP1*	del	0.000179	97	49
*XXBAC-B444P24*	del	5.97E-18	59	0	*TUBGCP5*	del	0.000179	97	49
*QPRT*	dup	1.98E-12	65	7	*CATG0024378*	del	0.00016	96	48
*KCTD13*	dup	3.53E-14	62	4	*BOLA2B*	dup	2.95E-11	51	4
*PAGR1*	dup	3.53E-14	62	4	*CATG0022287*	del	0.000191	94	47
*RTN4R*	del	1.17E-17	58	0	*AC023490*	del	4.04E-14	46	0

**Table 3:** The top 40 genes associated with Autism Spectrum Disorder (ASD) are presented in this study. These genes are identified as having the highest likelihood of causing ASD based on their variations.

Gene Name	Status	P-Value	CaseOV	ControlOV	Gene Name	Status	P-Value	CaseOV	ControlOV
*DGCR2*	del	2.38E-44	420	3	*GABRA5*	dup	9.82E-28	235	0
*ARVCF*	del	3.77E-44	418	3	*OCA2*	dup	8.85E-28	234	0
*GNB1L*	del	3.77E-44	418	3	*CATG00000022283*	del	5.01E-08	263	32
*CATG00000058206*	del	6.34E-44	417	3	*CATG00000022351*	dup	2.34E-27	231	0
*COMT*	del	6.34E-44	417	3	*NRXN1*	del	2.08E-12	249	19
*ZDHHC8*	del	6.34E-44	417	3	*LINC00624*	del	3.24E-15	241	13
*HIRA*	del	6.34E-44	417	3	*XXBAC-B135H6*	del	1.66E-21	229	4
*TBX1*	del	6.34E-44	417	3	*BCL9*	del	1.01E-16	234	10
*CDIPT*	del	4.15E-28	387	15	*CHD1L*	del	1.01E-16	234	10
*SEZ6L2*	del	4.15E-28	387	15	*FMO5*	del	1.01E-16	234	10
*ASPHD1*	del	4.15E-28	387	15	*ACP6*	del	1.01E-16	234	10
*KCTD13*	del	4.15E-28	387	15	*CATG00000092640*	del	5.84E-17	141	0
*CATG00000027072*	del	4.15E-28	387	15	*CATG00000058020*	del	1.96E-15	142	1
*CDIPT-AS1*	del	4.15E-28	387	15	*RFC2*	del	1.94E-15	141	1
*ALDOA*	del	4.04E-28	386	15	*WBSCR22*	del	9.53E-17	140	0
*FAM57B*	del	4.04E-28	386	15	*GTF2I*	del	9.53E-17	140	0
*CHRNA7*	del	1.75E-26	239	1	*STX1A*	del	9.53E-17	140	0
*DGCR5*	del	3.40E-21	244	6	*EIF4H*	del	1.94E-15	141	1
*GABRB3*	dup	9.82E-28	235	0	*DNAJC30*	del	9.53E-17	140	0
*OTUD7A*	del	2.64E-26	236	1	*VPS37D*	del	9.53E-17	140	0

**Table 4:** A contingency table was constructed to apply Fisher’s exact test. This table will be utilized in the analysis to calculate the p-value for the genes and DNA segments under investigation.

# of case samples overlapped with the gene	# of case samples not overlapped with the gene
# of control samples overlapped with the gene	# of control samples not overlapped with the gene

The study presents Tables 1, 2, and 3, which exhibit the top 40 genes for each disorder along with their corresponding p-values. Table 4 shows the formulation of Fisher’s exact test. CaseOV represents the number of overlaps between cases and the genes, while controlOV represents the number of overlaps between controls and the genes.

Furthermore, we examined the genes that are associated with all of the three disorders and those linked with only two of them. *COMT* deletion is common between ASD and SCZ, while deletions in *CYFIP1*, *PRODH*, *XXBAC-B444P24*, *LINC00896*, *ZDHHC8*, *AC006547*, *NIPA2*, *RTN4R*, *NIPA1*, and *TUBGCP5* are common between SCZ and DD. Next, common genes between ASD and DD are deletions in *FAM57B*, *SHANK3*, and *BDH1*, and the shared genes between the three disorders were deletions in *DGCR5* and *ZDHHC8*.

In the subsequent sections, a comparison was made with machine learning methods, followed by a search for genes displaying brain-enriched expression. Notably, it was observed that many genes associated with brain disorders possess brain-enriched functions [33]. We compared our results with similar studies, demonstrating that our research successfully identifies more brain-enriched genes than previous investigations.

Furthermore, we compare our findings to genes that cause nervous system phenotypes in mice, which were obtained from the MGI repository [34]. Our study demonstrates a higher fold enrichment than similar studies. The next step is identifying genotype-phenotype relationships using the DECIPHER data source [35], focusing on phenotypes exhibiting high enrichment levels.

In addition, we used WebGestalt [36] to perform gene ontology analyses of coding genes, with a focus on examining Gene Ontology (GO), Human Phenotype Ontology (HPO), and associated disease terms.

### Comparison with machine learning methods

Next, we compare our method with machine learning methods for the gene prioritization problem. The selected algorithms were guided backpropagation (GBP) [37], deepLIFT [38], and DeepGenePrior (without pre-training). The third choice is to show the effect of pre-training on the performance of the whole model (an ablation study). DeepLIFT [38] is a reference-based global feature importance algorithm that uses a correlation score to measure the input’s effect on the model’s output. Guided backpropagation is a global feature importance that is gradient-based.

The performance benchmarks are computed as follows. The model is trained comprehensively, and important genes are selected based on their respective weights. Subsequently, the model is retrained using the identified important genes as inputs and the disease status as the output. The performance evaluation is then reported based on the test set. Global methods mainly suffer from many computations and estimates (making the model inaccurate). DeepLIFT needs a reference for calculation; the reference is very influential in the final results of the model and may cause the model to choose the wrong inputs.

Guided backpropagation needs gradients, and it has been proven that the gradients can sometimes be noisy, resulting in the selection of irrelevant features. Other methods need several simple local surrogate models to interpolate the manifold in high-dimensional models (like LIME [39]); these surrogates impose massive calculations and imprecise the model.

Some advantages of our proposed method are that it does not need the reference, does not rely on noisy data, and is not local, and there is a way to inject unlabeled as well as labeled data in the model.

The Python torch Captum [40] implementations of these algorithms were used for the comparison.

**Fig 2:**
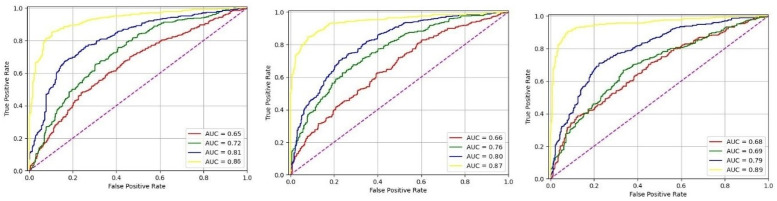
The Area Under the Curve (AUC) values for different machine learning methods. The yellow curve represents DeepGenePrior, the blue curve represents DeepGenePrior without Pretraining, the green curve represents Guided Backpropagation [37], and the Red is for DeepLIFT [38].

The results were reported in Table 5 regarding the accuracy and ROC AUC. Our DeepGenePrior algorithm Performs higher than the others. Besides, ROC curves are shown in Fig 2.

**Table 5:** A comparison was conducted with other machine learning methods to assess performance. The accuracies and ROC AUCs of various machine learning techniques were reported for three datasets. It was found that DeepGenePrior outperformed the other methods, demonstrating higher accuracy and ROC AUC values.

	SCZ		ASD		DD	
	Accuracy	ROC AUC	Accuracy	ROC AUC	Accuracy	ROC AUC
DeepGenePrior	80	.86	82	.87	83	.89
DeepGenePrior without Pretraining	74	.81	73	.80	71	.79
DeepLIFT [77]	60	.65	62	.66	63	.68
Guided Backpropagation [78]	65	.72	69	.76	64	.69

### Overrepresentation of tissue-specific genes

Several studies (such as [41] and [42]) claim that brain-enriched genes play an important role in BDs. To determine whether the detected genes are overrepresented in the brain tissue, we compute the fraction of coding and non-coding genes that have been enriched (background expectation) and compare it with the percentage of genes that have overlapped with deleted or duplicated CNVs.

The authors of [41] provide a list of brain-enriched genes. To obtain this list, they used the FANTOM5 CAGE-associated transcriptome [43] to identify coding and long non-coding RNA genes in the regions and examined their expression patterns across sample types.

In addition to alternative methods, we incorporated two gene prioritization tools, GeneFriends [44] and ToppGene [45], both accessible online. GeneFriends applies the guilt by association approach, while ToppGene identifies candidate genes based on functional similarity to the training gene list. However, these tools possess certain limitations. Notably, they have a restricted capacity for accommodating large datasets, require seed data for achieving results (following the guilt by association principle), and rely on parameter tuning by the user, such as setting a Pearson correlation threshold and an FDR threshold. For this analysis, the default parameter values were employed.

**Fig 3:**
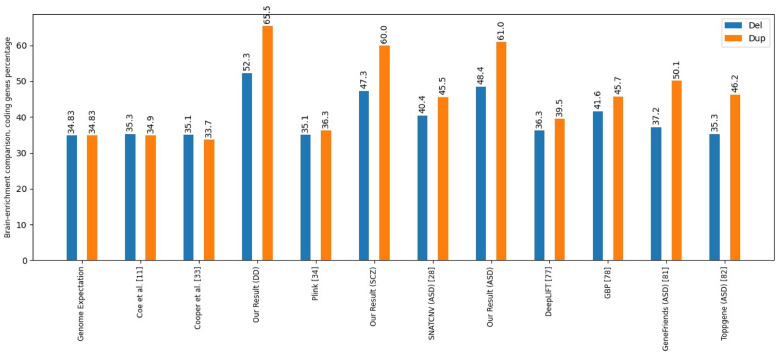
Brain-enrichment comparison, coding genes. This figure compares brain-enriched coding genes for different tools and methods. The percentage of brain-enriched coding genes was evaluated for two variation types, namely deletion, and duplication.

**Fig 4:**
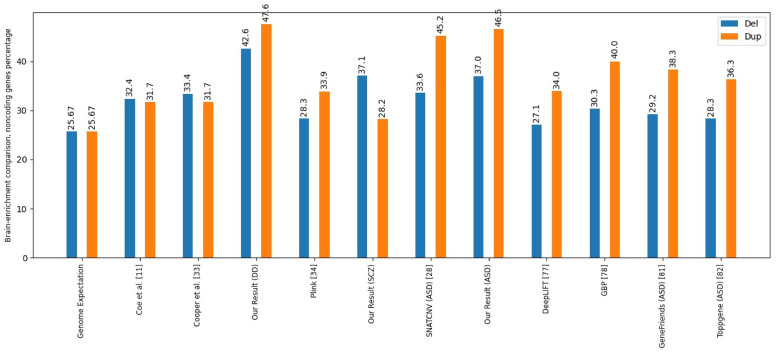
Brain-enrichment comparison, noncoding genes. This figure illustrates the comparison of different tools and papers based on the percentage of brain-enriched noncoding genes.

Fig 3 presents the results of brain-enriched coding genes fold enrichment, and Fig 4 illustrates brain-enriched lncRNA genes fold enrichment.

The results of our study are compared with those of Coe et al. [11] and Cooper et al. [46], two important studies of developmental delay. They were also compared with PLINK [47] and SNATCNV [41], publicly available tools with state-of-the-art performance.

In the list of brain-enriched genes related to ASD and SCZ, *DGCR2* specifies a protein proposed to be important in neural crest cell migration [30]. The *ZDHHC8* gene, strongly associated with ASD and SCZ [30], is another gene to note.

Next, we have some brain-enriched genes associated with SCZ; *RTN4R* is a gene in which adult central nervous systems are likely to be affected by its role in regulating axonal regeneration and plasticity. *CATG00000058203* and *Septin5* and *CATG00000057131* are some brain-enriched genes associated with ASD and SCZ, previously mentioned in [41].

As for the developmental delay, the *DGCR5*, *PRODH*, *NIPA1*, *TUBGCP5*, *RTN4R*, *ZDHHC8*, *CRKL*, and *SERPIND1* genes are also brain-enriched and associated with the disease. Most of them are from the 22^nd^ and 15^th^ chromosomes (22q11.21).

### Gene segregation analysis of male and female patients

Long-standing research shows that females are more tolerant of mutations than males, which explains why males are more prone to brain disorders such as autism. New studies also confirm the validity of previous findings [48–50] that male cases show more significant enrichment than female cases when comparing the ratios of cases to controls. In this research, we pointed out that some genes are more biased towards males, for example, deletion in *PHF2 (ENSG00000197724)*, duplication in *NRXN1 (ENSG00000179915)*, and deletions in *WDFY3 (ENSG00000163625)*, *PHF3 (ENSG00000118482)*, *MED13L (ENSG00000123066)*, and *WAC (ENSG00000095787)*, are more frequently seen in males than females for the developmental delay.

Besides, we performed the same analysis with ASD CNVs. We found that the *PTCHD1 (ENSG00000165186)* gene deletion occurred more in male than female patients. (Table 6 provides the details for these claims, and the chi-square test confirms the results).

**Table 6:** Gender Bias Analysis of the Brain Disorders. This table presents the Gender Bias Analysis of Brain Disorders, highlighting genes associated with one gender.

Gene Name	% Male Cases	% Male Controls	%Cases/ %Controls (Male)	% Female Cases	% Female Controls	%Cases/ %Controls (Female)	Log2 (Male/ Female Enrichment)
PTCHD1(Del) ♂	.29	.08	3.7	.11	.07	1.6	1.21
PHF2(Del) ♂	.05	.08	.62	.01	.07	.21	1.59
*NRXN1*(Dup) ♂	.07	.08	.87	.04	.14	.31	1.49
*WDFY3*(Del) ♂	.08	.08	.99	.03	.07	.41	1.27
*PHF3 (Del)* ♂	.98	.15	6.53	.45	.27	1.66	1.97
*MED13L(Del)* ♂	.73	.08	9.125	.17	.06	2.83	2.06
*WAC(Del)* ♂	1.2	.4	3	.21	.31	.677	2.15

### DECIPHER analysis

We used DECIPHER [35], the genotype-phenotype data source for almost 12,600 patients with CNVs, to analyze phenotypes associated with candidate genes.

**Fig 5:**
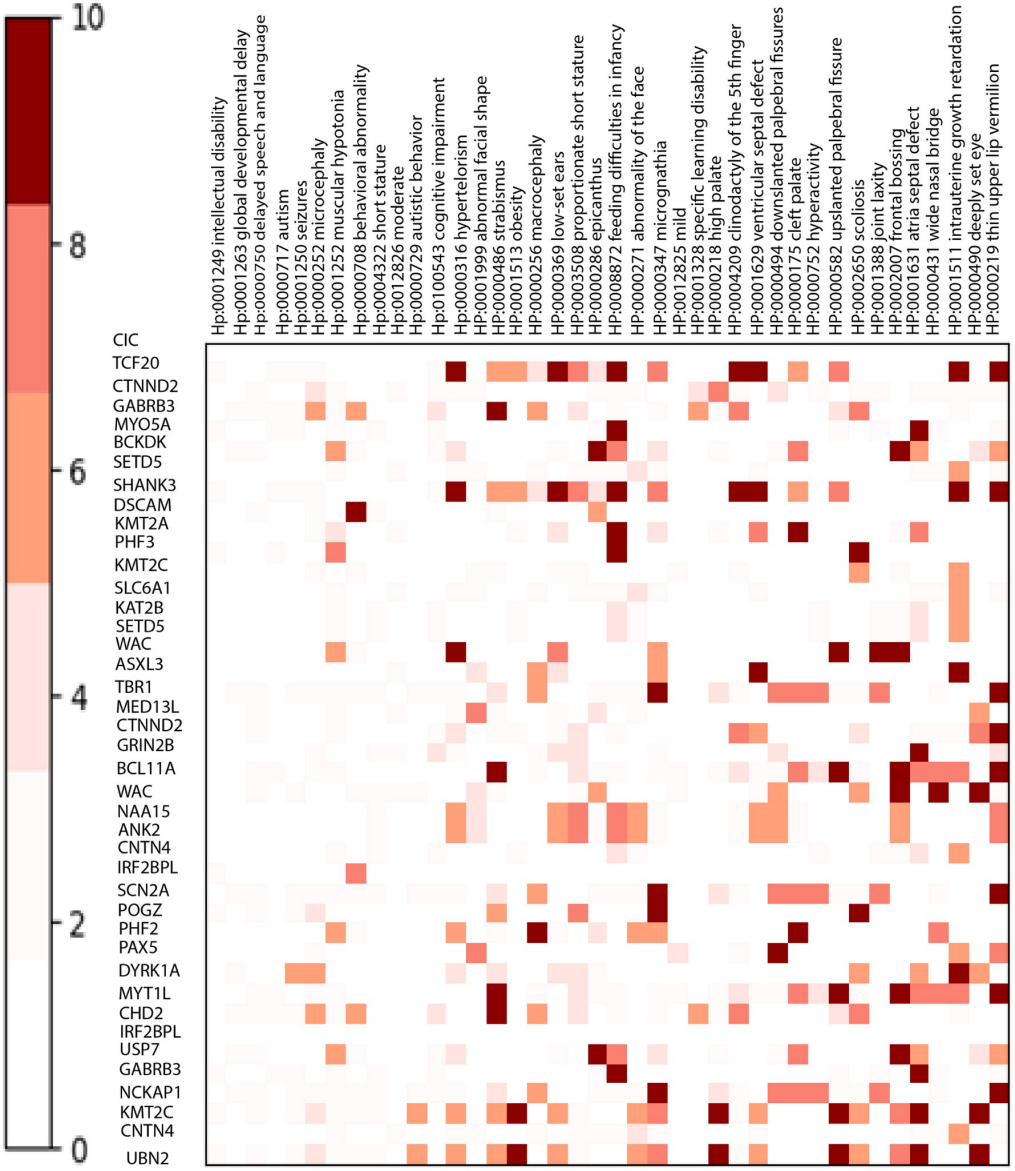
Heatmap for developmental delay. This figure showcases a Heatmap for Developmental Delay, providing insights into the relationship between candidate genes and DECIPHER phenotypes. The heatmap demonstrates a strong correlation between genes and phenotypes, depicted by the prominent dark red color.

To investigate the relationship between genes and phenotypes, we calculate the ratio of overlapped samples with the specific phenotype to the number of overlapped samples for a putative gene. Figs 5 (DD), 6 (SCZ), and 7 (ASD) depict the respective heatmaps for each target disorder.

**Fig 6:**
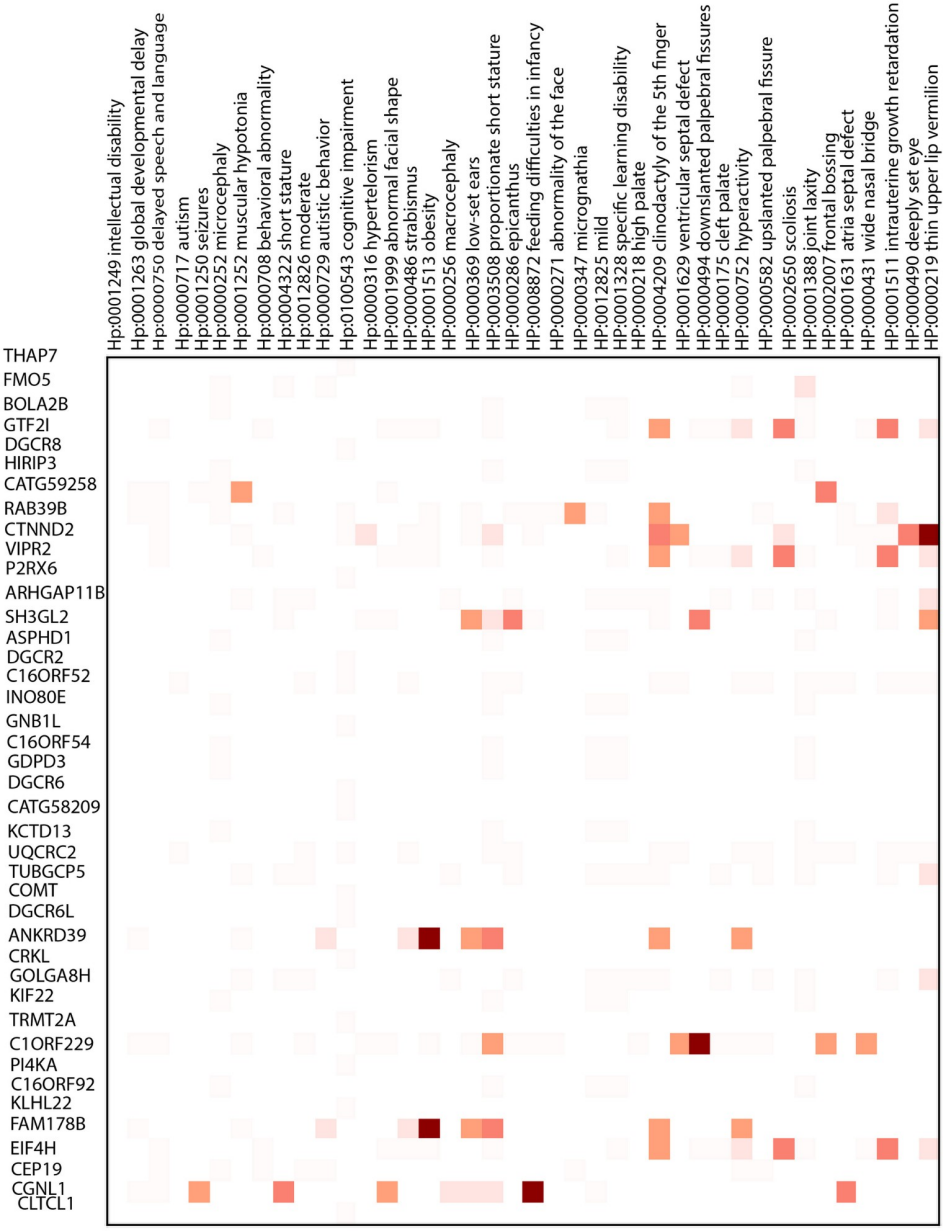
Heatmap for schizophrenia. This figure presents a Heatmap for Schizophrenia, where the horizontal labels represent genes associated with SCZ, and the vertical labels represent DECIPHER phenotypes. Detailed explanations of the results can be found in the accompanying text.

**Fig 7:**
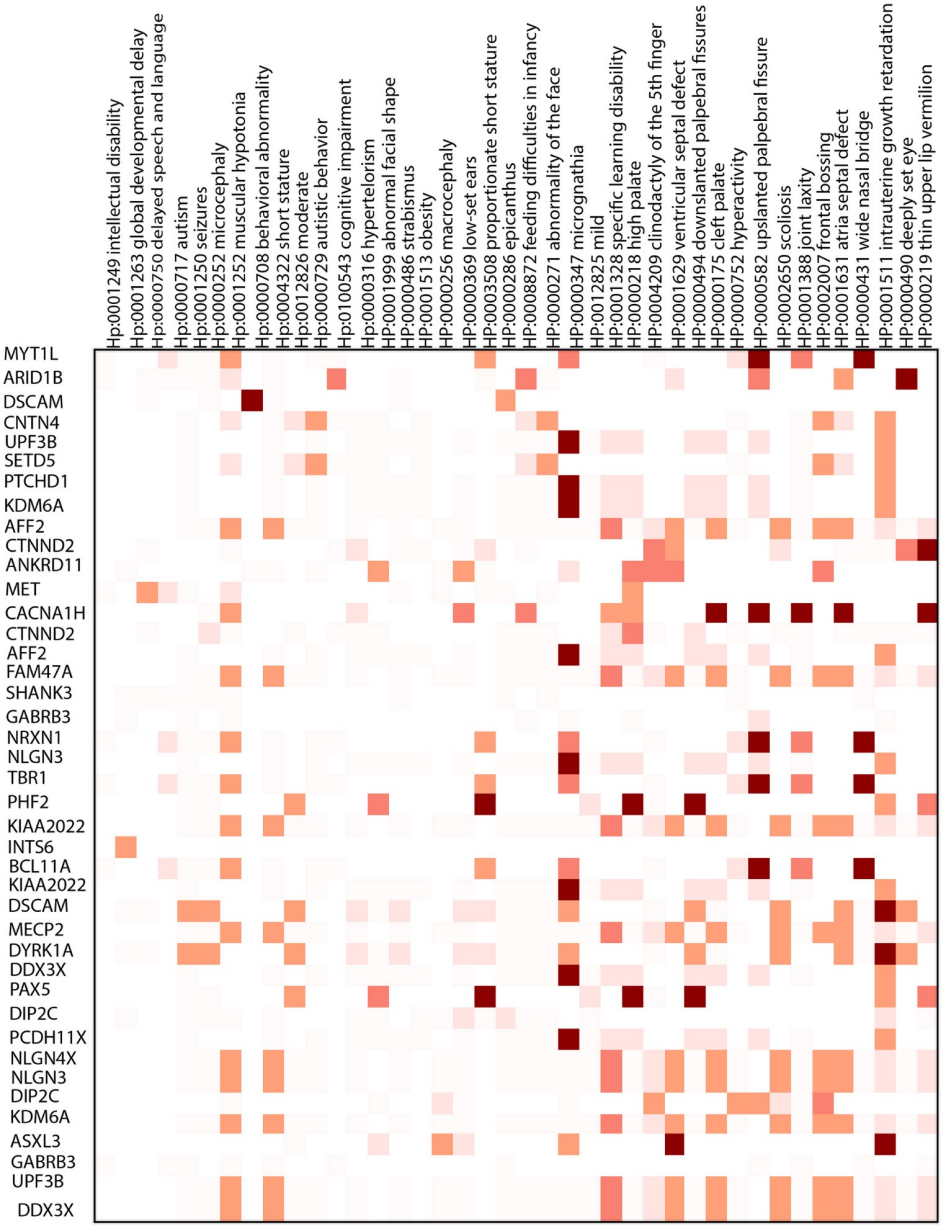
Heatmap for autism. The color legend is similar to the last heatmap.

Some of the highlighted phenotypes related to the target diseases are obesity (HP:0001513), autism (HP: 0000717), behavioral abnormality (HP: 0000708), irregularity of the face (HP: 00000271), and seizures (HP:0001250).

Children with autism are more likely to suffer from medical comorbidities. For example, we found macrocephaly (HP:00000256), hydrocephalus (HP:00000238), cerebral palsy (HP:0100021), migraine (HP:0002076), sleep disturbance (HP:0002360), and failure to thrive (HP:0001508) which was also mentioned in [51] as the phenotypes that co-occur in the autism. For schizophrenia, DECIPHER analysis revealed phenotypes such as obsessive-compulsive behavior (HP:0000722), anxiety (HP:0000739), and depression (HP:0000716), as well explained in [52]. *MVP* duplication, overrepresented in SCZ, is associated with depression (HP:0000716).

Regarding the developmental delay, secondary conditions such as microcephaly (HP:0000252) and anxiety (HP:0000739) can be proposed, which was also suggested in [53]; This disorder has received less research. *BCL9*, *FMO5*, and *GPR89B* deletions related to microcephaly are also overrepresented in DD. *NIPA1* duplication, associated with anxiety, is among the top genes of DD. In [53], microdeletion of the *NF1* gene is found to be associated with microcephaly and DD.

Our model deduces a set of genes for a target genetic disease. We investigated the set of phenotypes related to the genes; the specific relationship between genes and phenotypes shows that there can be diversity in the etiology of the disease, which implies that the occurrence of a phenotype in a target disease is influenced by what candidate genes are mutated in the patient.

### Analysis of biological processes and phenotypic ontologies of candidate genes

As part of our analysis, we used WebGestalt [36] to investigate the associations between identified genes and specific gene ontologies (GOs), human phenotype ontologies (HPOs), and disease terms [54,55].

Some examples of the discovered disease ontology terms were intellectual disability, language development disorders, poor school performance (for the developmental delay), autistic disorder, and language development disorders. Language development disorders are discussed in [56] as a comorbidity of BDs.

In the associated HPO terms, some examples were autistic behavior, delayed speech and language development, intellectual disability, severe global developmental delay, abnormal social behavior, impaired social interactions, and abnormally aggressive, impulsive, or violent behavior. Abnormal behavior is mentioned in [57], and impaired social interaction is discussed in [56] as phenotypes related to BDs.

The highlighted Gene Ontology terms include cognition, dendrite development, and synapse organization. In [58], dendrite development is pointed out to be associated with BDs, and the relationship between synapse organization and BDs is addressed in [59]. Tables 7, 8, and 9 summarize the results.

**Table 7:** WebGestalt Analysis for Developmental Delay. Three types of analyses were conducted for genes associated with developmental delay (DD). The table presents the p-value, false discovery rate (FDR), and the number of genes for each analyzed trait.

Description	p-value	FDR	#Genes	Type of Analysis
Autism spectrum disorders	4.02E-07	6.84E-04	7	Disease Ontology Terms
Intellectual disability	2.41E-06	2.05E-03	14	Disease Ontology Terms
Language development disorders	2.81E-05	1.60E-02	3	Disease Ontology Terms
Epileptic encephalopathy	7.90E-05	2.61E-02	4	Disease Ontology Terms
Mental retardation	1.38E-04	2.61E-02	11	Disease Ontology Terms
Low intelligence	1.38E-04	2.61E-02	11	Disease Ontology Terms
Mental deficiency	1.38E-04	2.61E-02	11	Disease Ontology Terms
Poor school performance	1.38E-04	2.61E-02	11	Disease Ontology Terms
Dull intelligence	1.38E-04	2.61E-02	11	Disease Ontology Terms
Cognition	1.03E-05	8.29E-03	8	Gene Ontology
Intraspecies interaction between organisms	8.84E-05	2.36E-02	4	Gene Ontology
Covalent chromatin modification	9.89E-05	2.36E-02	7	Gene Ontology
Chemical synaptic transmission, postsynaptic	1.44E-04	2.36E-02	5	Gene Ontology
Regulation of membrane potential	1.72E-04	2.36E-02	8	Gene Ontology
Multi-organism behavior	1.76E-04	2.36E-02	4	Gene Ontology
Neuron projection guidance	2.34E-04	2.44E-02	6	Gene Ontology
Dendrite development	2.43E-04	2.44E-02	6	Gene Ontology
Neuron projection organization	5.50E-04	4.91E-02	4	Gene Ontology

**Table 8:** WebGestalt Analysis for Schizophrenia. The table displays the results of the WebGestalt analysis conducted for schizophrenia. Several traits have demonstrated significant correlations with brain disorders, as determined by various types of analysis.

Description	P-Value	FDR	#Genes	Type of Analysis
Blepharophimosis	4.04E-07	4.29E-04	5	Disease Ontology Terms
Hernia, Inguinal	7.50E-07	4.29E-04	5	Disease Ontology Terms
Chronic otitis media	1.01E-06	4.29E-04	4	Disease Ontology Terms
ear infection chronic	1.01E-06	4.29E-04	4	Disease Ontology Terms
Proteinuria	8.52E-06	9.68E-04	5	Disease Ontology Terms
Redundant skin	9.10E-06	9.68E-04	3	Disease Ontology Terms
Bunion	9.10E-06	9.68E-04	3	Disease Ontology Terms
Hallux Valgus	9.10E-06	9.68E-04	3	Disease Ontology Terms
Sloping shoulders	9.10E-06	9.68E-04	3	Disease Ontology Terms
Congenital anomaly of neck	9.10E-06	9.68E-04	3	Disease Ontology Terms
Deformity of neck	9.10E-06	9.68E-04	3	Disease Ontology Terms
Malformation of the neck	9.10E-06	9.68E-04	3	Disease Ontology Terms
Hypoplastic toenails	9.10E-06	9.68E-04	3	Disease Ontology Terms
Phonophobia	9.10E-06	9.68E-04	3	Disease Ontology Terms
Colon diverticulum anatomic structure	9.10E-06	9.68E-04	3	Disease Ontology Terms
Diverticular disease of the colon	9.10E-06	9.68E-04	3	Disease Ontology Terms
Pointed chin	1.05E-05	1.05E-03	4	Disease Ontology Terms
Sacral dimples	1.81E-05	1.62E-03	3	Disease Ontology Terms
Pulmonary Stenosis	2.30E-05	1.62E-03	4	Disease Ontology Terms
Prominent lower lip	2.89E-05	1.62E-03	4	Disease Ontology Terms
Posterior embryotoxic	4.02E-08	3.95E-05	6	Human Phenotype Ontology
Abnormality of the line of Schwalbe	4.02E-08	3.95E-05	6	Human Phenotype Ontology
Retinal vascular tortuosity	4.02E-08	3.95E-05	6	Human Phenotype Ontology
Abnormal systemic arterial morphology	6.88E-08	5.06E-05	10	Human Phenotype Ontology
Retinal arteriolar tortuosity	4.40E-07	2.59E-04	4	Human Phenotype Ontology
Abnormal aortic morphology	5.50E-07	2.70E-04	8	Human Phenotype Ontology
Abnormal concentration of calcium in the blood	6.93E-07	2.92E-04	6	Human Phenotype Ontology
Patellar dislocation	5.94E-06	1.82E-03	4	Human Phenotype Ontology
Multiple renal cysts	5.94E-06	1.82E-03	4	Human Phenotype Ontology
Tetralogy of Fallot	7.50E-06	1.82E-03	6	Human Phenotype Ontology
Abnormality of calcium homeostasis	7.50E-06	1.82E-03	6	Human Phenotype Ontology
Blepharophimosis	8.79E-06	1.82E-03	6	Human Phenotype Ontology
Conotruncal defect	8.79E-06	1.82E-03	6	Human Phenotype Ontology
Inguinal hernia	8.82E-06	1.82E-03	7	Human Phenotype Ontology
Myocardial infarction	9.25E-06	1.82E-03	4	Human Phenotype Ontology
Atrophy/Degeneration involving the corticospinal tracts	1.37E-05	2.25E-03	4	Human Phenotype Ontology
Abnormality of divalent inorganic cation homeostasis	1.38E-05	2.25E-03	6	Human Phenotype Ontology
Abnormal ventriculoarterial connection	1.38E-05	2.25E-03	6	Human Phenotype Ontology
Abnormal connection of the cardiac segments	1.59E-05	2.46E-03	6	Human Phenotype Ontology
Cholelithiasis	1.97E-05	2.63E-03	4	Human Phenotype Ontology

**Table 9:** WebGestalt analysis for autism spectrum disorder.

Description	P-value	FDR	#Genes	Type of Analysis
Autism Spectrum Disorders	3.73E-08	6.34E-05	7	Disease Ontology Terms
Autistic Disorder	1.36E-06	1.16E-03	9	Disease Ontology Terms
Language Development Disorders	1.07E-05	6.10E-03	3	Disease Ontology Terms
Intellectual Disability	9.44E-05	3.89E-02	10	Disease Ontology Terms
Autistic behavior	1.14E-04	3.89E-02	3	Disease Ontology Terms
Intraspecies Interaction Between Organisms	8.79E-09	7.07E-06	6	Gene Ontology Terms
Multi-Organism Behavior	2.62E-08	1.05E-05	6	Gene Ontology Terms
Cognition	4.24E-07	1.14E-04	8	Gene Ontology Terms
Chemical Synaptic Transmission, Postsynaptic	9.33E-07	1.87E-04	6	Gene Ontology Terms
Dendrite Development	2.54E-05	4.08E-03	6	Gene Ontology Terms
Adult Behavior	3.05E-05	4.09E-03	5	Gene Ontology Terms
Membrane Biogenesis	6.32E-05	7.22E-03	3	Gene Ontology Terms
Synapse Organization	7.18E-05	7.22E-03	7	Gene Ontology Terms
Regulation Of Membrane Potential	8.37E-05	7.48E-03	7	Gene Ontology Terms
Respiratory Gaseous Exchange	2.21E-04	1.71E-02	3	Gene Ontology Terms
Peptidyl-Lysine Modification	2.33E-04	1.71E-02	5	Gene Ontology Terms
Neuron Projection Guidance	3.09E-04	2.07E-02	3	Gene Ontology Terms
Regulation Of Neurological System Process	4.82E-04	2.98E-02	4	Gene Ontology Terms
Covalent Chromatin Modification	8.51E-04	4.89E-02	5	Gene Ontology Terms

### Overrepresentation of homologs of coding genes causative of nervous system phenotypes in the mutated mouse

Studying animal genetic mutations provides insight into disease mechanisms and treatments for brain disorders. Several animal models have been developed to uncover the disorder’s process [60]. Mutant mice with specific defects in the nervous system are among them. Models based on mutant mice replicate key symptoms of brain disorders.

We investigate what percentage of the causative genes have homologs in mouse genes whose mutation causes nervous system phenotypes. For this purpose, we used the Mouse Genome Informatics (MGI) database to identify genes related to the mouse nervous system and their human homologs.

**Fig 8:**
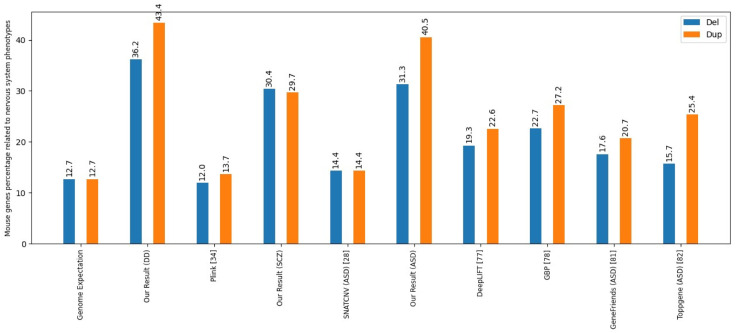
Percentage of Mouse Genes Associated with Nervous System Phenotypes. The figure compares results obtained from various tools and methods, categorized according to types of variation. The analysis focuses on the proportion of mice genes contributing to nervous system phenotypes.

Fig 8 presents an analysis of the proportions of homologs among the identified genes exhibiting nervous system phenotypes in mice. The findings reveal that the coding genes identified by our method show a higher percentage of homologs in mutant mouse models displaying nervous system phenotypes compared to the results obtained from other methods. We also evaluated two gene prioritization tools, GeneFriends [44] and ToppGene [45]. For example, some genes that have orthologs in mice with nervous system phenotypes are *SEPTIN5*, *RTN4R*, and *ZDHHC8*. These genes are common among the three disorders.

### Statistical analysis

**Fig 9:**
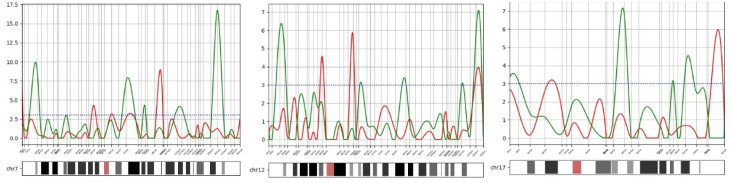
Distribution of -log10(p-value) across Chromosomes with Deletion and Duplication Samples. The figure displays the distribution of -log10(p-value) using Fisher’s exact test for deletion (green curves) and duplication (red curves). The horizontal axis represents the chromosome loci, while the vertical axis represents the -log10(p-value).

Subsequently, an independent statistical analysis is conducted to compare the outcomes of DeepGenePrior with hypothesis testing performed in similar studies. Sample results are depicted in Fig 9. To assess whether the observed associations are statistically significant or occur by chance, 100,000 random permutations of case and control labels were performed. The corresponding results are illustrated in the respective diagrams.

## Discussion

In this paper, we presented a deep learning approach that uses a variational autoencoder to analyze CNVs and prioritize genes within them systematically. Our deep learning model learns how features are distributed over samples, which enables us to predict the likelihood that a gene variation will cause a specific disease. We applied our method to three disorders under the umbrella term of brain disorders. We examined the results for overrepresentation of enriched brain coding, long non-coding RNA genes, and mouse orthologs with nervous system phenotypes. Additionally, we used the DECIPHER data to investigate how variations in the identified genes influence other traits. Furthermore, we conducted gene ontology analyses.

We analyzed 118,968 case CNVs from 48,748 patients and 76,528 control CNVs from 26,063 healthy individuals for gene associations with brain disorders. Among the top 40 genes associated with developmental delay, *DGCR6*, *PRODH*, *DGCR5*, and *ZDHHC8* are potential candidates for involvement in DiGeorge syndrome pathology and schizophrenia. Additionally, the expression of *MVP* may serve as a prognostic marker for several types of cancer. For schizophrenia, *DGCR6* and *PRODH* are well-known candidate genes, and *DGCR5* is a long non-coding RNA gene with a high score for causing schizophrenia. Furthermore, *SEZ6L2*, *CDIPTOSP*, *ASPHD1*, and *RANBP1* are potential candidate genes for schizophrenia. For autism spectrum disorder, *DGCR2*, *ARVCF*, *GNB1L*, *COMT*, *ZDHHC8*, *CHRNA7*, and *NRXN1* are candidate genes with various associated developmental defects.

Schizophrenia is a complex and debilitating mental disorder associated with genetic factors. One of the well-known candidate genes for schizophrenia is *DGCR6*, which codes for a protein. In addition, mutations in *PRODH* (Proline Dehydrogenase 1), located on 22q11.21, have been linked with susceptibility to schizophrenia (*SCZD4*). Another potential genetic factor for schizophrenia is *DGCR5*, which is a long non-coding RNA (lncRNA) with a high score for causing schizophrenia. [30]

*SEZ6L2* (Seizure Related 6 Homolog Like 2), located in 16p11.2, is another gene implicated in mental disorders. This region is thought to hold candidate genes for autism spectrum disorder. *CDIPTOSP* (CDIP Transferase Opposite Strand, Pseudogene) is a lncRNA gene associated with Central Nervous System Germ Cell Tumor disease. *ASPHD1* (Aspartate Beta-Hydroxylase Domain Containing 1) is another gene linked with schizophrenia (specifically, Schizophrenia 3). Lastly, *RANBP1* (RAN Binding Protein 1) is a protein-coding gene linked with Digeorge Syndrome.

Autism spectrum disorder (ASD) is a complex developmental disorder linked to genetic factors. One such factor is the deletion of *DGCR2*, which has been associated with a wide range of developmental defects. These defects are collectively called *CATCH 22*, which stands for DiGeorge syndrome, velocardiofacial syndrome, conotruncal anomaly face syndrome, and isolated conotruncal cardiac defects. Additionally, the *ARVCF* gene is responsible for autosomal dominant Velo-Cardio-Facial syndrome (VCFS), which is characterized by cleft palate, conotruncal heart defects, and facial dysmorphology. *GNB1L* is another gene that is deleted in DiGeorge syndrome. [61,62]

Schizophrenia and panic disorder are two other mental disorders that have been linked to genetic factors. One such factor is the *COMT* (Catechol-O-Methyltransferase) gene, which codes for a protein and has been associated with both schizophrenia and Panic Disorder 1. Another gene linked to schizophrenia is *ZDHHC8* (Zinc Finger DHHC-Type Palmitoyltransferase 8), which is located on chromosome 6q24-q25.

We also investigated the gender distribution of CNVs in BDs. We found that duplication in *NRXN1* and deletion in *PTCHD1* are more frequently observed in males than females for some of the BDs.

We observed that some brain-enriched coding genes were significantly expressed in all three disorders. Examples include *DGCR2*, *SEPTIN5*, and *ARVCF*, which are located on chromosome 22 and have a deletion associated with these disorders. These three genes were among the top ten coding brain-enriched genes related to the three disorders. We also found that *DGCR5*, a noncoding brain-enriched gene previously known as a biomarker for Huntington’s disease, is highly associated with DD. *AC000068* is a noncoding brain-enriched gene associated with SCZ and ASD. *SEPTIN5* has been previously shown to be associated with ASD and SCZ, while *DGCR2* is mainly known to be associated with SCZ. *AC004471* is a noncoding brain gene among the top 10 genes related to SCZ, ASD, and DD.

Among the top genes with significant brain expression, some have orthologs in mice that showed nervous system phenotypes. *SEPTIN5*, *ZDHHC8*, *RTN4R*, and *KCTD13* are the top genes for ASD and SCZ, while *RTN4R* and *ZDHHC8* also rank highly in DD. *SEZ6L2* is top in ASD but has a lower rank in SCZ. *ZDHHC8* and *RTN4R* are genes with nervous system morphological and physiological phenotypes, while *SEPTIN5* shows only nervous and physiological phenotypes in mice.

In the next step, we used DECIPHER [35] to examine the relationship between the detected genes and other phenotypes. We found that delayed speech, language, and autism were associated with the set of genes. According to our findings, seizures were associated with SCZ; this relationship was previously discussed in [63].

Microcephaly [64] and macrocephaly [65] are two reverse phenotypes associated with ASD and SCZ. Additionally, ’abnormal facial shape’ is associated with all three disorders [66], which has also been studied in [67]. *CACNA1H* is one of the genes related to some overrepresented phenotypes [68], and *TCF20*, discussed in [69], is another gene highlighted in the heatmap of developmental delay.

We performed gene ontology analysis for the detected genes using the WebGestalt tool. This tool allowed us to perform gene ontology analysis, human phenotype ontology (HPO) analysis, and disease ontology analysis separately. For the disease ontology, some terms were “Language Development Disorders,” “Autistic behavior,” and “Congenital neck anomaly.” Overrepresented HPO terms included “Severe global developmental delay,” “abnormal social behavior,” “Delayed speech and language development,” and “Intellectual disability.” Some of the most common gene ontology terms were “dendrite development,” “cognition,” and “Regulation of Neurological System Process.” In summary, these findings support the biological relevance of the method-identified genes to genetic factors that contribute to brain disorders.

Although the application of our model focused on three specific brain disorders, it is important to note that our method is not limited to these disorders alone. The versatility of our approach allows for its application in any case-control study involving copy number variants associated with different target disorders. Consequently, the method inherently generates a list of candidate genes specific to the target disorder.

In future research, we plan to explore integrating network analysis techniques and combining mutational data with other auxiliary information, such as proteins or other modalities. This integration will enable the utilization of various modeling tools, such as graphs, to uncover additional patterns within the mutational data.

## Materials and methods

### Data and preprocessing

In this study, we analyzed three case-control datasets comprising individuals with brain disorders, namely autism spectrum disorder, schizophrenia, and developmental delay. After preprocessing and quality control, the autism spectrum disorder dataset consisted of 47,119 cases and 24,858 control copy number variants (CNVs), as documented in the AUTDB database [41]. The schizophrenia dataset comprised 42,046 cases and 40,414 control CNVs [70]. The developmental delay dataset included 29,803 cases and 11,256 control CNVs. These datasets were selected based on their relevance to the genetic etiology of brain disorders and the availability of reliable and well-curated CNV data.

The final data source for developmental delay comprised two independent datasets with two different data types: NSTD 54 [46] and NSTD 100 [11]. We utilized the NSTD 100 dataset, which includes gender data. All CNVs in this dataset are rare, with a frequency of less than 1% of the population. Further details regarding these CNVs are reported in Table 10.

**Table 10:** Statistics of different datasets. The number of case and control individuals, along with the number of CNVs, were reported in the table.

Dataset	of case CNVs	of control CNVs	Sum	Ratio	of Patients	of Healthy	Sum	Ratio
Autism spectrum disorder	47,119	24,858	71,977	~1.89	19,663	6,479	26,142	~3.03
Schizophrenia	42,046	40,414	82,460	~1.05	28,684*	28,893*	57,577	~0.99
Developmental delay (NSTD 100)	29,803	11,256	41,059	~2.64	29,085	19,584	48,669	~1.52

* 13k Affy and 15k Illumina for cases, 14K Affy, and 14k Illumina for controls.

We used two supplementary data sources in our study. The first is the FANTOM5 (Functional Annotation of the Mammalian Genome 5) Atlas [71], which includes 21,069 protein-coding and 27,920 non-coding genes.

The second data source we used is the Database of Chromosomal Imbalance and Phenotype in Humans Using ENSEMBL Resources (DECIPHER, February 1st, 2017) [35]. This dataset contains information on patients, CNVs, and phenotypes such as ASD, DD, and SCZ. We investigate DECIPHER website to analyze the relationship between genes and other phenotypes and to augment and pretrain our system. Table 11 shows the statistics of the dataset.

**Table 11:** DECIPHER statistics [35]. DECIPHER is a genotype-phenotype data source that can be used to investigate the associations between genes and traits.

**Num of Patients**	~12,600 Patients	
**Num of CNVs**	~16,600 CNVs	
**Num of Phenotypes**	~2,615 Phenotypes	
**Num of Autism Patients**	~ 1,548 Patients	Related Phenotypes: Autism, Autistic behavior, Autism with high cognitive abilities
**Num of Developmental Delay Patients**	~ 2,144 Patients	Related Phenotypes: Global developmental delay, Mild global developmental delay, severe global developmental delay, Neurodevelopmental delay, Moderate global developmental delay
**Num of Schizophrenia Patients**	~ 12 Patients	Related Phenotypes: Schizophrenia, Schizencephaly

In DECIPHER, there were 1,548 patients with ASD-related phenotypes, including ’HP:0000717’ (autism), ’HP:0000729’ (autistic behavior), and ’HP:0000753’ (autism with high cognitive abilities). The dataset also contained 2,144 patients with DD-related phenotypes, including ’HP:0001263’ (global developmental delay), ’HP:0011342’ (mild global developmental delay), ’HP:0011344’ (severe global developmental delay), ’HP:0011343’ (moderate global developmental delay), and ’HP:0012758’ (neurodevelopmental delay).

This paper also analyzed tissue-enriched genes with a high brain expression level compared to other tissues. We utilized the list of brain-enriched genes provided in [41]. While [42] highlights the impact of brain-enriched genes on autism spectrum disorder, our study focuses on their effect on schizophrenia and developmental delay.

Additionally, we used MGI (Mouse Genome Informatics) data [34] to determine if candidate genes related to disease cause a nervous system phenotype in mice, following a similar approach as [41]. HTML was parsed from pages covering nervous system phenotype (MP:0003631) [72], abnormal nervous system morphology (MP:0003632) [73], and abnormal nervous system physiology (MP:0003633) [74]. The mapping was performed using [75]. The data preprocessing involved CNV filtering, conversion, and supplementary data cleansing (DECIPHER data analysis, FANTOM5 data, etc.).

For CNV filtering and conversion, we filtered out CNVs smaller than one kbps (similar to other studies such as [11,41,46]). The CNV studies also had different coordinates (hg17, hg18, and hg19). Therefore, we unified all CNVs and converted them to hg19 using the UCSC Lift Genome Annotations tools [31]. Moreover, we removed Y chromosome CNVs due to insufficient data, eliminating all CNVs with missing values.

We removed patients without phenotypes during supplementary data cleansing while using the DECIPHER data. Preprocessing was unnecessary for Fantom5, MGI, and brain genes since all gene coordinates were already in the hg19 format and ready for processing.

Furthermore, we removed some genes that were not the result of the model, such as genes that overlapped more with controls than cases or genes that did not overlap with CNVs.

### A formal overview of a gene prioritization system

In the context of gene prioritization, the process can be conceptualized as a system where the input consists of a target disease and a comprehensive gene list. Depending on the methodology employed for gene processing, various additional datasets may also be incorporated as auxiliary input. These datasets could include protein networks, pathway data, or reliable candidate genes associated with the target disease, thereby leveraging the "guilt by association" principle. The desired output is a list of candidate genes, which can be sorted or unsorted, representing the outcome of prioritization or classification. Furthermore, a scoring system may be implemented to indicate the likelihood of a gene’s association with a particular phenotype or disease. The discriminatory algorithm aims to infer the role of each gene in the development of the target disease.

This section aims to provide a formal definition of our work. Consider a case-control study about a specific target disease. This study comprises copy number variants observed in both patient and healthy control groups. The CNVs can be defined as quadruples, characterized as follows:

$$CNV_{set}=\left\{\left(ch;dosage;strt;stp\right)|ch\;in\;\left\{1..24\right\};dosage\;in\;\left\{del;dup\right\};strt<stp\right\}.$$ (1)

where ch is the chromosome number, the dosage is the type of CNV, either deletion or duplication, and strt and stp determine the region of the chromosome where the variation occurs. The CNVs are for people (specified by an identifier) whose features (like gender, other phenotypes, etc.) may or may not be available.

This CNV is available in two sets: one for cases and one for controls.


$$D_{input}=D_{case}\cup D_{control}$$ (2)

$$D_{case\left|control=\left\{{(p}_{id};CNV_{set}\right)\right|p_{id}\;is\;the\;id\;of\;an\;individual;CNV_{set}is\;the\;rare\;CNVs\;for\;him\}}$$ (3)

Each rare CNV is related to an individual (characterized by p_id), either healthy or patient. Additionally, the dataset may optionally include auxiliary data for each individual, such as gender information. This supplementary information enables us to explore the discriminatory role of genes for each gender. Our objective is to address the gene prioritization problem utilizing a set of rare copy number variants.

### The method overview

Compared to conventional machine learning methods, deep learning approaches offer the advantage of constructing a feature hierarchy and reducing data dimensions. This facilitates the identification of hidden patterns within the data more effectively than alternative approaches. An example of deep learning is the autoencoder, which plays a crucial role in dimensionality reduction and generating a concise, high-level representation of the data through a hierarchical arrangement of features [6]. The autoencoder consists of an encoder network (inference network) that progressively transforms the input into a low-dimensional latent representation and a decoder network (generative network) that strives to reconstruct the output to closely resemble the original input. Autoencoders have been widely employed in various bioinformatics problems [76–78].

**Fig 10:**
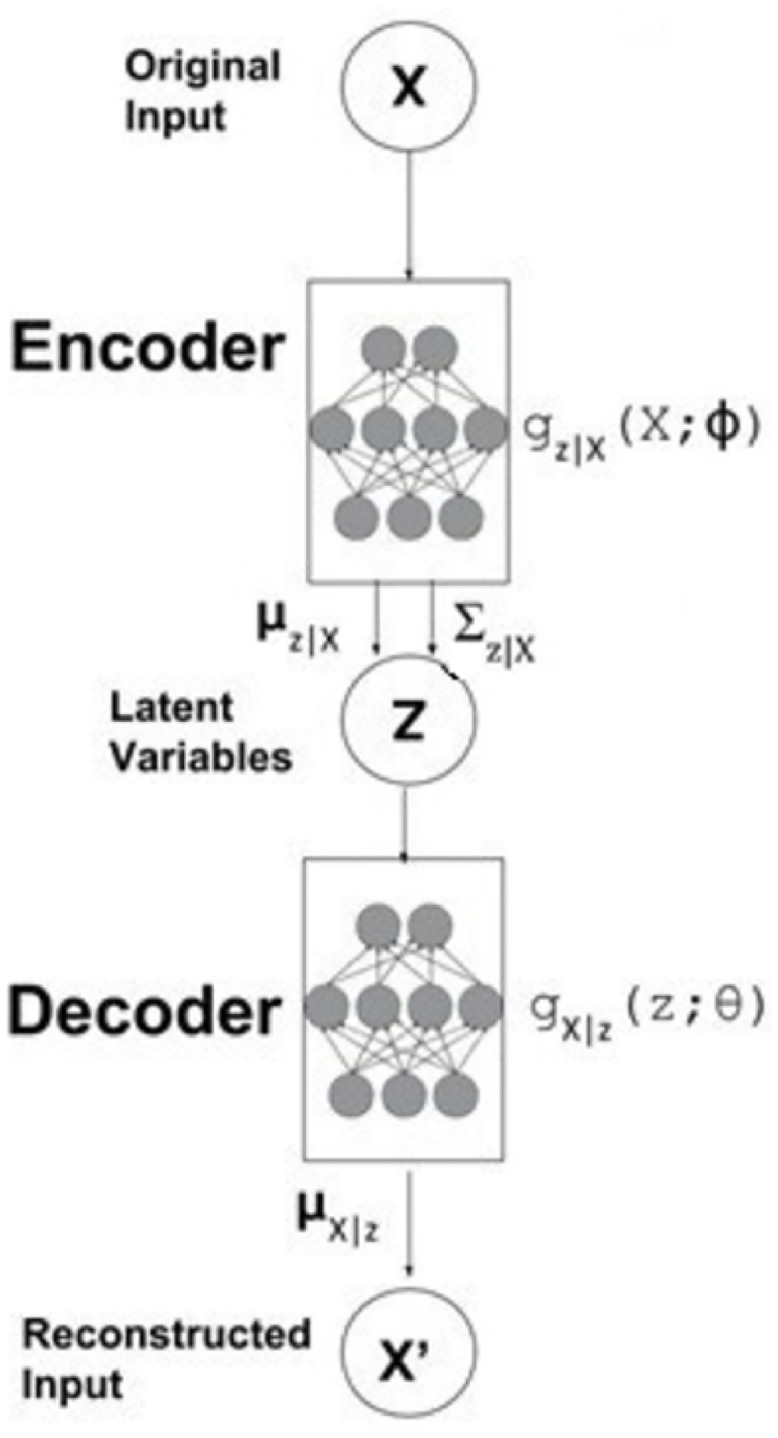
Visualization of a Two-Step Semisupervised Variational Autoencoder (VAE) Process. The figure illustrates the two steps involved in training the VAE. Initially, the VAE is trained in an unsupervised manner. In the second step, one part of the VAE is utilized for training with labels, introducing supervised learning. [80].

Combining autoencoders with the variational learning framework results in the development of Variational Autoencoders VAE [28,79]. VAEs further enhance the capabilities of autoencoders. Fig 10 presents an overview of the VAE, illustrating its schematic representation.

The primary distinction between autoencoders and their variational counterpart lies in their inherent nature. Autoencoders operate deterministically, while variational autoencoders (VAEs) adopt a probabilistic approach. VAEs, in particular, employ regularization techniques to prevent overfitting during training. VAEs are founded upon the Bayesian theorem and inference principles, incorporating a regularization constraint. This framework assumes that the latent representation follows a multivariate Gaussian distribution, denoted as N(μ, σ).

Numerous studies have demonstrated that VAE exhibits enhanced stability during training and produces less ambiguous outputs than other generative models. This improved performance can be attributed to VAE’s optimization of precise objective functions rooted in likelihood principles [81]. The posterior distribution in VAE is modeled as a Gaussian distribution, characterized by its mean and variance. It has been theoretically proven that this Gaussian distribution can approximate any function effectively. The primary objective of the VAE model is to encode the input data into a Gaussian distribution, estimating its mean and covariance.

VAE, a deep generative model that utilizes variational inference, is designed to discover a low-dimensional latent representation, denoted as z, for high-dimensional input data X, following the probability distribution P(X). To capture the intrinsic information of the input dataset, P(z|X), the estimation of the posterior distribution becomes necessary, which is typically intractable. By employing variational inference, a distribution family Q(z|X) (referred to as the variational distribution) is introduced to approximate the P(z|X) distribution. The objective is to minimize the Kullback-Leibler (KL) divergence (D) between these two distributions, serving as a dissimilarity measure.


$$D[Q(z|X)|\left|P\left(z|X\right)\right]=E_{z\sim Q}[\text{log}\;Q\left(z|X\right)-\text{log}\;P(z|X)].$$ (4)

After some calculations, we have the following objective function, which is the variational lower bound on log-likelihood:

$$\begin{array}{l} \text{log}\;p_{\theta }\left(x\right)\geq l_{VAE}\\ =\text{log}\;P\left(X\right)-D[Q(z|X)||P\left(z|X\right)]\\ =E_{z\sim Q}[\text{log}\;P(X|z)]-D(Q(z|X)||P\left(z\right)] \end{array}$$ (5)

The first term is the expectation over the approximate posterior distribution (named as reconstruction error), and the second term (KL distance) is the regularizer (P (z) is standard Gaussian Distribution, N(0, I)). Q(z|X) is the encoding distribution, and P (X|z) is the decoding distribution.

Utilizing these equations transforms the minimization task into a maximization task. The encoder, denoted as Q(z|X), and the decoder, denoted as P(X|z), play crucial roles in this process. This goal can be achieved using deep neural networks coupled with stochastic gradient variational Bayes. In the VAE framework, the encoder component is employed to generate the parameters of the variational distribution. To mitigate overfitting, the dropout technique can be applied. The recognition model Q(z|X) takes the form of a multi-dimensional Gaussian distribution, where the network generates the mean and covariance of this Gaussian distribution. As for the latent space, a standard Gaussian distribution N(0, I) is employed as the prior distribution.

The loss function in VAE comprises two terms: the reconstruction loss, which facilitates efficient encoding and decoding similar to an autoencoder, and the regularization term, also known as the latent loss, which imposes constraints on the latent space. The regularization term approximates the latent space to follow a standard Gaussian distribution. To incorporate the regularization, the VAE loss function incorporates the Kullback-Leibler divergence, which encourages the covariance matrix to be close to the identity matrix and the mean to be zero.

The training process of the deep learning models consists of two phases: pretraining and fine-tuning. During the pretraining phase, the autoencoder is trained to learn high-level features from all the CNVs associated with the disorders. In the subsequent fine-tuning step, the decoder is set aside, and only the dedicated CNVs specific to the target disease are utilized for training.

### The method details

**Fig 11:**
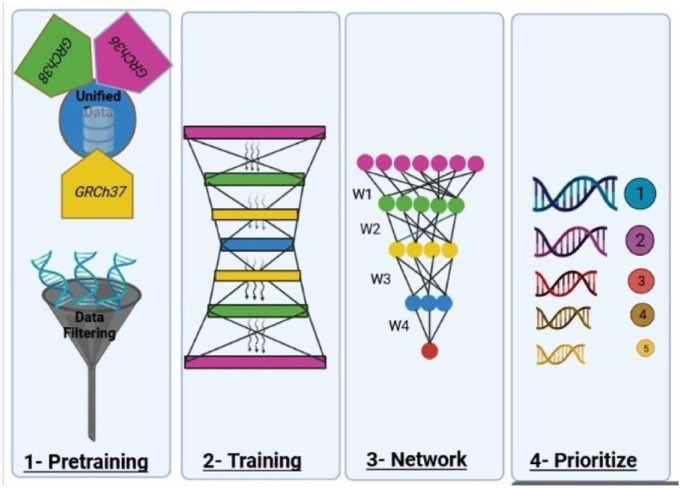
Overview of the Proposed Method. This figure presents a schematic representation of the entire process involved in the proposed method. The process consists of several sequential steps, as illustrated from left to right. The first step involves data preprocessing for data preparation. Initially, the data is obtained in various formats such as hg18, hg19, etc. To establish uniformity, the data is converted into a unified format, specifically hg19. Additionally, this step eliminates redundant, useless, and incomplete features from the data. In the second step, a model is constructed using the cleaned data. This model takes the form of an autoencoder. Subsequently, the weights of the network are adjusted using the corresponding labels. These labels assign values of zero or one to distinguish between healthy and patient individuals. In the fourth step, the autoencoder’s coefficients are utilized to prioritize the genes. The importance of each gene is represented by the size of its corresponding icon in the figure. Larger icons correspond to more significant genes, whereas smaller icons indicate less important genes.

In this section, we explain our method for prioritizing genes. An overview of the method is provided in Fig 11.

**Fig 12:**
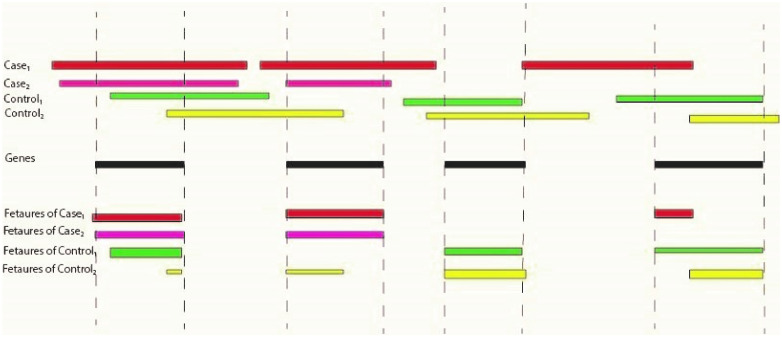
Features Generated from Cases and Controls. This figure presents the features derived from a group of cases and controls. Specifically, the figure depicts two cases and two controls, along with four genes of interest. The copy number variants (CNVs) observed in patients and healthy individuals are visually represented as rectangles in the top section of the figure. Furthermore, the overlaps, which signify the values of features for each case and control, are illustrated in the bottom section. These overlaps provide insight into the shared characteristics between the cases and controls.

A deep learning model is proposed for this task. According to the dataset for each disease, we have a copy number of variants for patients and healthy individuals. Each set of copy number variants for an individual has some overlaps with genes, which are features that feed into our deep learning. This is shown in Fig 12.

We have a list of genes that we want to determine whether their expression will affect disease incidence; besides, we have a list of cases and controls with CNVs for a target disease. We want to convert them to a supervised learning algorithm.

We need to convert CNVs to genes for each healthy and patient individual. Computing overlaps can do this. For the set of genes preprocessed, as discussed before, we measure the length of overlap (in kbps) with the CNVs of an individual. The label of the training set is whether the person is healthy or patient (zero or one).

In the pretraining phase of the model, we used all the CNVs of the brain disorders (autism + schizophrenia + developmental delay). In the next stage, fine-tuning, the CNV of a specific disease is used. Thus, here we have used semi-supervised learning.

After our VAE has been fully trained, we just use the encoder part directly for the next step:

1. Train a VAE using all our data points and transform our data (X) into the latent space (Z variables) (We use all data in this step).
2. Solve a standard supervised learning problem with (Z, Y) pairs (Y is the label set).

**Fig 13:**
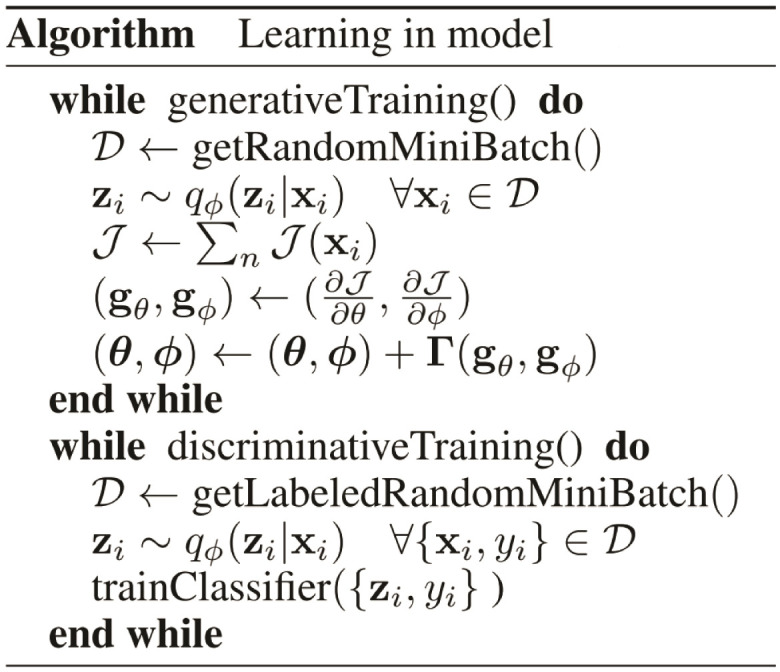
Variational autoencoder(VAE), the learning process algorithm [82].

The learning algorithm for the whole process is shown in Fig 13. In this algorithm, p is the true posterior, q is the approximate posterior distribution, z is the latent variable, θ is the decoder (z|x) parameters (generative model), and φ is the encoder (x|z) parameters (inference model).

Let’s suppose that the encoder weights are represented by $W_{i\times j}^{m}$, where m is the layer number, i is the output size in the last layer, and j is the input size in the current layer (no connection is determined by zero). As we know, the final layer that will be attached to the encoder is the label; and its size is one (whether the individual is patient (= one) or healthy (= zero)).

If we multiply all weights matrices together, the result has the size input size × 1 (the matrices are multiplicable since the output of the last layer equals the input of the next layer). The resulting matrix (precisely column vector) can rank genes according to the label (the label is the status of the disease), and this is the same thing we want to model. The formulation is as follows:

$$W_{I\times 1}^{final}=W_{I\times .}^{1}\times W^{2}\times \ldots \times W_{.\times 1}^{M}$$ (6)

The specification of the deep learning model is such that a binary classification task is accomplished. The size of each layer is the square root of the number of nodes of the last layer. The final layer has a binary outcome, the last activation function is sigmoid, and loss function is binary cross-entropy, and the optimization algorithm is Adam.

Additionally, we investigate the novelty of the top ten genes in three disorders by conducting a comprehensive literature search (searching the ’gene name’ + ’disorder name,’ the gene will be labeled as known if a meaningful result is obtained. Table 12 shows the results of this investigation.

**Table 12:** Top ten genes suggested to be candidates for brain disorders and their status in last publications.

	Gene Name	Lastly Discovered
Developmental Delay	*TDRP*	Novel
	*DGCR5*	Novel
	*PRODH*	Novel
	*LCE3E*	Novel
	*ERICH1*	Known
	*CATG0101427*	Novel
	*ERICH1-AS1*	Novel
	*RP11-462G22*	Novel
	*CATG0074892*	Novel
	*CATG0074890*	Novel
Schizophrenia	*CATG0074891*	Novel
	*DGCR6*	Known
	*PRODH*	Known
	*DGCR5*	Known
	*AC009133*	Novel
	*MVP*	Novel
	*CDIPT*	Known
	*SEZ6L2*	Known
	*CATG0027072*	Novel
	*CDIPT-AS1*	Novel
Autism Spectrum Disorder	*DGCR2*	Known
	*ARVCF*	Known
	*GNB1L*	Known
	*CATG00000058206*	Novel
	*COMT*	Known
	*ZDHHC8*	Known
	*HIRA*	Novel
	*TBX1*	Known
	*CDIPT*	Known
	*SEZ6L2*	Known

### The detail of the implementation

The deep learning model has a training phase, which needs a training set including cases and controls. We developed the system with Python and PyTorch [83]. We used cross-validation and grid search to tune the parameters (like the number of neurons in each layer).

The activation functions are empirically selected Rectified Linear Units, and the weights were optimized by an adaptive optimization algorithm (Adam) [84] to minimize reconstruction error and loss. The decoder has a symmetrical structure to the encoder. The learning rate, decay rate, and epoch were set to 0.001 and 1, and at most 10,000, respectively. Also, we restrict connections to some extent for a reduction in parameters. The train/test ratio is set to 80/20. The number of layers is at most three.

## Supporting information

S1 Table Details of the results for Autism Spectrum Disorder. (XLSX)

S2 Table Details of the results for Schizophrenia. (XLSX)

S3 Table Details of the results for Developmental Delay. (XLSX)

S1 Fig The common genes between disorders, ‘del’ is short for deletion. (EPS)

S2 Fig Distribution of CNV length in different chromosomes for SCZ disease; y − Axis is the ×10^5^. The numbers on top of the plot show the number of cases and controls. The red color (left) represents cases, and the blue (right) represents controls.
(EPS)

S3 Fig Distribution of CNV length in different chromosomes for ASD disease. Y-Axis is the ×10^6^. The numbers on top of the plot show the number of cases and controls. The red color (left) represents cases, and the blue (right) represents controls.
(EPS)

S4 Fig Distribution of CNV length in different chromosomes for DD disease. Y-Axis is the ×10^6^. The numbers on top of the plot show the number of cases and controls. The red color (left) represents cases, and the blue (right) represents controls.
(EPS)

S5 Fig Demographic Distribution of DD and ASD datasets. (EPS)

S6 Fig Decipher Phenotypes Frequency. (EPS)

S7 Fig Details of the setup of the method. Since the technique is semisupervised, the first step is to use the data without labels to pretrain the network. The next step is to use the target data to fine-tune it. Next, we use the coefficients of the network to derive a score for each of the features of the input network, i.e., genes. The values of the scores are then sorted so that the relative usefulness of the genes can be evaluated.
(EPS)
